# The Effect of Handwashing at Recommended Times with Water Alone and With Soap on Child Diarrhea in Rural Bangladesh: An Observational Study

**DOI:** 10.1371/journal.pmed.1001052

**Published:** 2011-06-28

**Authors:** Stephen P. Luby, Amal K. Halder, Tarique Huda, Leanne Unicomb, Richard B. Johnston

**Affiliations:** 1 International Centre for Diarrhoeal Disease Research, Bangladesh, Dhaka, Bangladesh; 2 Centers for Disease Control and Prevention, Atlanta, Georgia, United States of America; 3 Water and Environmental Sanitation Section, UNICEF Bangladesh, Dhaka, Bangladesh; Aga Khan University, Pakistan

## Abstract

**Background:**

Standard public health interventions to improve hand hygiene in communities with high levels of child mortality encourage community residents to wash their hands with soap at five separate key times, a recommendation that would require mothers living in impoverished households to typically wash hands with soap more than ten times per day. We analyzed data from households that received no intervention in a large prospective project evaluation to assess the relationship between observed handwashing behavior and subsequent diarrhea.

**Methods and Findings:**

Fieldworkers conducted a 5-hour structured observation and a cross-sectional survey in 347 households from 50 villages across rural Bangladesh in 2007. For the subsequent 2 years, a trained community resident visited each of the enrolled households every month and collected information on the occurrence of diarrhea in the preceding 48 hours among household residents under the age of 5 years. Compared with children living in households where persons prepared food without washing their hands, children living in households where the food preparer washed at least one hand with water only (odds ratio [OR] = 0.78; 95% confidence interval [CI] = 0.57–1.05), washed both hands with water only (OR = 0.67; 95% CI = 0.51–0.89), or washed at least one hand with soap (OR = 0.30; 95% CI = 0.19–0.47) had less diarrhea. In households where residents washed at least one hand with soap after defecation, children had less diarrhea (OR = 0.45; 95% CI = 0.26–0.77). There was no significant association between handwashing with or without soap before feeding a child, before eating, or after cleaning a child's anus who defecated and subsequent child diarrhea.

**Conclusions:**

These observations suggest that handwashing before preparing food is a particularly important opportunity to prevent childhood diarrhea, and that handwashing with water alone can significantly reduce childhood diarrhea.

*Please see later in the article for the Editors' Summary*

## Abstract 2

By observing handwashing behavior in 347 households from 50 villages across rural Bangladesh in 2007, Stephen Luby and colleagues found that hand washing with soap or hand rinsing without soap before food preparation can both reduce the burden of childhood diarrhea.

## Editors' Summary

**Background:**

The resurgence of donor interest in regarding water and sanitation as fundamental public health issues has been a welcome step forward and will do much to improve the health of the 1.1 billion people world-wide without access to clean water and the 2.4 billion without access to improved sanitation. However, improving hygiene practices is also very important—studies have consistently shown that handwashing with soap reduces childhood diarrheal disease—but in reality is particularly difficult to do as this activity involves complex behavioral changes. Therefore although public health programs in communities with high child mortality commonly promote handwashing with soap, this practice is still uncommon and washing hands with water only is still common practice—partly because of the high cost of soap relative to income, the risk that conveniently placed soap would be stolen or wasted, and the inconvenience of fetching soap.

Handwashing promotion programs often focus on five “key times” for handwashing with soap—after defecation, after handling child feces or cleaning a child's anus, before preparing food, before feeding a child, and before eating—which would require requesting busy impoverished mothers to wash their hands with soap more than ten times a day.

**Why Was This Study Done?:**

In addition to encouraging handwashing only at the most critical times, clarifying whether handwashing with water alone, a behavior that is seemingly much easier for people to practice, but for which there is little evidence, may be a way forward. In order to guide more focused and evidence-based recommendations, the researchers evaluated the control group of a large handwashing, hygiene/sanitation, and water quality improvement program—Sanitation, Hygiene Education and Water supply-Bangladesh (SHEWA-B), organized and supported by the Bangladesh Government, UNICEF, and the UK's Department for International Development. The researchers analyzed the relationship between handwashing behavior as observed at baseline and the subsequent experience of child diarrhea in participating households to identify which specific handwashing behaviors were associated with less diarrhea in young children.

**What Did the Researchers Do and Find?:**

The SHEWA-B intervention targeted 19.6 million people in rural Bangladesh in 68 subdistricts. In this study and with community and household consent, the researchers organized trained field workers, using a pretested instrument, to note handwashing behavior at key times and recorded handwashing behavior of all observed household at baseline in 50 randomly selected villages that served as nonintervention control households to compare with outcomes to communities receiving the SHEWA-B program. The fieldworkers recruited community monitors, female village residents who completed 3 days training on how to administer the monthly diarrhea survey, to record the frequency of diarrhea in children aged less than 3 years in control households for the subsequent two years. The researchers used statistical models to evaluate the association between the exposure variables (household characteristics and observed handwashing) and diarrhea.

Using these methods, the researchers found that compared to no handwashing at all before food preparation, children living in households where the food preparer washed at least one hand with water only, washed both hands with water only, or washed at least one hand with soap, had less diarrhea with odds ratios (ORs) of 0.78, 0.67, and 0.19, respectively. In households where residents washed at least one hand with soap after defecation, children had less diarrhea (OR = 0.45), but there was no significant association between handwashing with or without soap before feeding a child, before eating, or after cleaning a child's anus, and subsequent child diarrhea.

**What Do These Findings Mean?:**

These findings from 50 villages across rural Bangladesh where fecal environmental contamination, undernutrition, and diarrhea are common, suggest that handwashing before preparing food is a particularly important opportunity to prevent childhood diarrhea, and also that handwashing with water alone can significantly reduce childhood diarrhea. In contrast to current standard recommendations, these results suggest that promoting handwashing exclusively with soap may be unwarranted. Handwashing with water alone might be seen as a step on the handwashing ladder: handwashing with water is good; handwashing with soap is better. Therefore, handwashing promotion programs in rural Bangladesh should not attempt to modify handwashing behavior at all five key times, but rather, should focus primarily on handwashing after defecation and before food preparation. Furthermore, research to develop and evaluate handwashing messages that account for the limited time and soap supplies available for low-income families, and are focused on those behaviors where there is the strongest evidence for a health benefit could help identify more effective strategies.

**Additional Information:**

Please access these Web sites via the online version of this summary at http://dx.doi.org/10.1371/journal.pmed.1001052.

- A four-part collection of Policy Forum articles published in November 2010 in *PLoS Medicine*, called “Water and Sanitation,” provides information on water, sanitation, and hygiene
- Hygiene Central provides information on improving hygiene practices

## Introduction

Intervention trials promoting handwashing with soap in communities with high child mortality consistently report a reduction in childhood diarrheal disease [1]. Bolstered by these data, public health programs serving low-income populations commonly promote handwashing with soap. Across a wide range of low-income countries, however, handwashing with soap is uncommon. In a review of structured observations in 11 countries mothers of young children washed their hands with soap on average only 17% of the time after using the toilet [2]. Barriers to washing hands with soap in low-income communities include the high cost of soap relative to household income, the risk that soap left out in a convenient place would be wasted by children playing with it or stolen, and the time required and inconvenience of fetching soap [3],[4]. In contrast to the low frequency of handwashing with soap, handwashing with water alone is more commonly practiced. In the same 11 countries study, mothers washed their hands with water alone 45% of the time after toileting [2]. Because intervention trials of handwashing with soap consistently demonstrated a health benefit, handwashing promotion interventions focus almost exclusively on handwashing with soap [5].

A second common characteristic of handwashing promotion programs is a focus on five “key times” for handwashing. These include handwashing after defecation, after handling child feces or cleaning a child's anus who had defecated, before preparing food, before feeding a child, and before eating [5],[6]. Asking mothers of young children to wash their hands with soap after each of these critical times would typically translate into requesting busy impoverished mothers to wash their hands with soap more than ten times a day. If mothers also follow recommendations to wash hands after touching domestic animals, animal dung, potentially contaminated raw food, and after coughing or sneezing [7], the number of recommended times for handwashing with soap would often exceed 20 times per day. In low-income households, soap is used judiciously to preserve money for food and other essentials [8]. Washing hands with soap this frequently, especially if practiced by all family members, would affect household finances. In order to preserve the household supply of soap, families commonly store soap away from the most convenient place to wash hands [3]. Washing hands with soap 10 or more times per day also takes a lot of time, time that mothers in low-income settings do not have in abundance [9]. The time required for handwashing with soap is especially onerous if lathering is continued for the full recommended 20 seconds [10] and soap is not kept at the most convenient place to wash hands. A third barrier to promoting handwashing at five different key times is the complexity of the message. A critical review of health communication interventions in low-income countries concluded that interventions that focus on a few messages were more effective than communication interventions targeting many behaviors [11].

Two steps that might improve the effectiveness of handwashing promotion interventions would be to encourage handwashing only at the most critical times for interrupting pathogen transmission and clarifying whether handwashing with water alone, a behavior that is apparently much easier for people to practice, should be encouraged. There are however, few data available to guide more focused recommendations.

In 2007, the Government of Bangladesh Department of Public Health Engineering in collaboration with UNICEF and with support from the Department for International Development (DFID) of the British Government launched a program, Sanitation, Hygiene Education and Water supply-Bangladesh (SHEWA-B) that is among the largest intensive handwashing, hygiene/sanitation, and water quality improvement programs ever attempted in a low-income country. The intervention targeted 20 million people in rural Bangladesh. As part of the assessment of the program's impact, fieldworkers conducted household structured observations at baseline in 50 randomly selected villages that served as nonintervention control households to compare with outcomes to communities receiving the SHEWA-B program. Community monitors assessed the frequency of diarrhea in control households each month for the subsequent 2 y. We analyzed the relationship between handwashing behavior as observed at baseline and the subsequent experience of child diarrhea in these households. The objective of this analysis was to identify which specific handwashing behaviors were associated with less diarrhea.

## Methods

### Ethics Statement

UNICEF publicly requested bids for the evaluation of the SHEWA-B program. The International Centre for Diarrhoeal Disease Research, Bangladesh (ICDDR,B) was selected through a competitive process and signed a contract with UNICEF for the evaluation. From UNICEF's perspective, and the perspective of the Government of Bangladesh, this was not a research contract. It was a contract to evaluate a US$90 million program targeting 20 million people across rural Bangladesh. The Government of Bangladesh separately contracted with 58 organizations to implement the intervention across 68 rural subdistricts on an aggressive launch schedule. The program evaluation required a preintervention baseline survey. If the evaluation team postponed field work for the 12- or more wk process that is characteristic for local human study participant protocol review and approval, ICDDR,B would have been unable to provide a preintervention measurement. This would have reduced the ability of ICDDR,B to assess the program, and would represent a failure to meet contractual obligations. We received ICCDR,B administrative approval to classify this activity as a nonresearch program evaluation that did not require independent human study participant review because the primary goal of this activity, particularly from the sponsor's perspective, was program evaluation and not generation of new generalizable knowledge.

The plan for the evaluation was reviewed by UNICEF and the Government of Bangladesh Department of Public Health Engineering, but was not reviewed by an independent human study participant committee. Each field worker received formal training in taking informed consent. As part of the consent process the field worker clarified how much time we were requesting from prospective participants. Field workers explained that there was no individual benefit or compensation for participation, that there would be questions about use of water, toilet facilities, and handwashing, and noted that these topics may be uncomfortable or that it may be uncomfortable to have a stranger in their home observing household activities. Twice during the consent process the field workers specified that participation was voluntary. They explained that even if the participant originally provided consent, he or she could withdraw consent at any time. Field workers secured written informed consent from each participant. Fields workers provided participants with contact information for the study coordinator and the research administration office of ICDDR,B if they had any questions. All collected information was kept in locked rooms. Only staff whose responsibilities included working with the data had access to the data. Study supervisors made unannounced visits to field teams to ensure that field workers properly implemented the enrollment and consent process.

### Study Population

The SHEWA-B program targeted 68 subdistricts (*upazilas*) in 19 districts. The government and UNICEF selected the specific intervention *upazilas* because of the perceived need and the absence of other active programs addressing water, sanitation, and hygiene in these communities. *Upazilas* are further subdivided into unions. We listed all of the unions and their populations in the 68 targeted subdistricts and randomly selected 50 unions with the probability of selection proportional to the size of the union. For each SHEWA-B intervention *upazila* where a union was chosen for evaluation we selected a control *upazila* that had similar geography, hydrogeology, infrastructure, agricultural productivity, and household construction, and where the government confirmed that no other major water-sanitation-hygiene programs were ongoing. We selected unions for evaluation in the control *upazilas* using the same probability of selection proportional to size used to select unions for evaluation in the intervention upazilas.

Within each selected union we listed all village names, and used a random number generator to select the evaluation village. Fieldworkers asked residents of the selected village to identify the village center. Fieldworkers identified the household closest to the village center that had a child <5 y of age and sought consent to permit a fieldworker to observe household practices for a single morning. To enroll the next household, fieldworkers looked for the next closest household with a child <5 y of age. Fieldworkers repeated the process for enrolling additional households until ten households in each selected village were enrolled for structured observation. Households enrolled for structured observation that had children <3 y of age, and so would remain <5 y of age during 2 y of follow-up, were also invited to participate in monthly disease surveillance.

### Data Collection

#### Structured observation

Trained fieldworkers conducted a single 5-h structured observation of handwashing behavior of all persons in selected households between 9:00 AM and 2:00 PM, a culturally acceptable time for visitors and a typical time period for a range of personal hygiene and food preparation behaviors. Using a pretested instrument, fieldworkers noted handwashing behavior at key times—before preparing food, eating or feeding a child, and after defecating or cleaning a child's anus who had defecated. Fieldworkers recorded handwashing behavior of all observed household residents because multiple persons commonly perform various caretaking roles and have contact with young children and all of these interactions may transmit diarrheal pathogens.

When observing food preparation, fieldworkers classified the food preparer's hands as contaminated if at least one hand contacted raw food or soil. Once hands were contaminated, fieldworkers noted whether or not the food preparer washed her hands before touching food. For preparing a single food item, fieldworkers often observed multiple opportunities for the preparers' contaminated hands to touch food. The fieldworkers recorded the most commonly observed handwashing behavior following contamination for each food item prepared.

#### Cross-sectional survey

Two months after the structured observation, fieldworkers returned to the households and administered a cross-sectional survey. Questions included on the cross-sectional survey included demographic information as well as household construction and possessions to permit a measurement of acquired household wealth.

#### Monthly surveillance

Fieldworkers recruited one woman (a community monitor) from each evaluation community who was at least 18 y of age and who had completed at least 8 y of formal education to visit each of the enrolled households each month and administer a brief questionnaire to collect information on each child <5 y of age. These community monitors participated in a formal training program to learn how to administer the questionnaire. The initial 3-d training included 2 d of classroom instruction with role playing followed by 1 d of field testing. After 12 mo the community monitors participated in a 1-d refresher training session. One of the monthly surveillance questions asked whether the child had diarrhea during the preceding 2 d. Another question asked if the child consumed only breast milk in the preceding 24 h. This surveillance continued for 24 mo following the cross-sectional survey. The community monitors were paid a modest stipend.

### Data Analysis

#### Exposure categories

Drawing water from a hand pump to wash hands or pouring water from a pitcher requires one hand. Rural Bangladeshi residents commonly run water over the “dirty hand” to clean it. If they use soap they sometimes roll the soap within the one hand. For each of the five key handwashing opportunities we classified the handwashing behavior into one of five categories: (1) no observed handwashing, (2) washing one hand with water alone, (3) washing both hands with water alone, (4) washing at least one hand with ash or mud, and (5) washing at least one hand with soap. We selected these categories because across the five key handwashing opportunities these categories displayed a range of handwashing behaviors that generally included sufficient observations to support analysis of associations.

Within each household, during the 5 h of observation fieldworkers often observed multiple occasions of the same opportunity for handwashing. For example, because there may be multiple children in the household and multiple snacks in addition to the main meal, the fieldworkers might observe four opportunities to wash hands before feeding a child. The handwashing behavior might be different in each of these four episodes, and fieldworkers recorded each episode separately. We classified the thoroughness of handwashing along a scale: the least thorough handwashing was no handwashing, washing one hand with only water, washing both hands with only water, washing with mud/ash, and washing with soap were progressively classified as more thorough handwashing. The household's handwashing behavior for each key time was classified on the basis of the most thorough handwashing behavior observed.

We considered drying hands with a clean towel or allowing hands to air dry before touching another surface as optimal hand drying. We classified drying hands on a visibly dirty towel, drying hands on clothing, or not drying hands before touching another surface, as progressively less optimal hand drying. We classified the household's hand drying behavior for each handwashing opportunity on the basis of the most optimal hand drying behavior observed at that key time.

#### Household wealth

We used principal component analysis of 21 household characteristics (Table 1) to evaluate household wealth [12]. We excluded hygiene and sanitary infrastructure, because we wanted to analyze the impact of wealth independent of the specific facilities that might contribute to handwashing. We analyzed variables in the wealth index by means or frequencies and calculated score coefficients. We used the correlation matrix of the 21 variables to calculate sample weights [13]. We calculated the coefficients by rounding the expression (Loading/standard deviation)×100 to the nearest integer. We used the first principal component as the wealth score [14].

**Table 1:** Characteristics of participating households, rural Bangladesh 2007.

Characteristic	All Participating Households (*n* = 347)		Households with Defecation Noted during Structured Observation (*n* = 102)	
	*n*	Percent or Mean	*n*	Percent or Mean
**General**				
Number household residents	1,891	5.4	603	5.9
Number of children age <5 y	379	1.1	144	1.4
Mean age (mo) of children <5 y	379	19.2	114	18.0
Father of the youngest child lacked formal education	118	34%	33	33%
Mother of the youngest child lacked formal education	96	28%	27	26%
**Occupation of father of the youngest child^a^**				
Laborer	82	24%	26	25%
Farmer/rickshaw puller or homemaker	116	33%	39	38%
Skilled worker	29	8%	7	7%
Working abroad	27	8%	10	10%
Salaried employee	39	11%	5	5%
Business owner	48	14%	13	13%
**Drinking water source**				
Shallow tube well	280	81%	82	80%
Deep tube well	25	7%	8	8%
Tara pump	15	4%	2	2%
Piped water	10	3%	1	1%
Protected well	9	3%	3	3%
Other	8	2%	6	6%
Owned source of drinking water	97	28%	41	40%
Owned toilet	175	50%	62	61%
Used improved latrine	264	76%	80	78%
**Proportion who owned**				
House^a^	324	94%	93	91%
Wardrobe^a^	106	31%	38	37%
Bicycle^a^	94	27%	32	31%
Mobile phone^a^	87	25%	28	27%
Black and white television^a^	67	19%	29	28%
Color television^a^	41	12%	13	13%
Sewing machine^a^	22	6%	9	9%
Refrigerator	10	3%	3	3%
Motor cycle	5	1%	1	1%
**Mean number of items owned**				
Tables^a^	347	1.1	102	1.2
Chairs^a^	347	2.3	102	2.9
Watches/clocks^a^	347	1.5	102	1.9
Beds^a^	347	0.9	102	1.2
Inexpensive sleeping cots^a^	347	1.2	102	1.4
**Acres of agricultural land^a^**	347	1.05	102	1.65
**Acres of nonagricultural land^a^**	347	0.22	102	0.23
**House construction**				
Tin roof^a^	309	89%	91	89%
Cement floor^a^	31	9%	17	17%
Brick walls^a^	32	9%	15	15%
Mean number of rooms^a^	347	2.2	102	2.4
Electrical connection*	169	49%	48	47%
**Cooking fuel^a^**				
Crop residue/grass	193	56%	53	52%
Wood	94	27%	28	27%
Dung	59	17%	20	20%

a Items used to construct the wealth index.

#### Modeling the handwashing–diarrhea relationship

We calculated odds ratios to evaluate the association between the exposure variables—household characteristics and observed handwashing—and diarrhea. To account for the repeated observations for diarrhea in single households and the clustering of observations in villages we used general estimated equations to calculate these adjusted odds ratios and 95% confidence intervals [15].

We constructed a multivariate model for each of the key times when handwashing behavior was significantly associated (*p*<0.05) with diarrhea in the bivariate analysis. We began with a bivariate model that included handwashing behavior, and sequentially added each of the household characteristics that were associated with diarrhea in bivariate analyses. The final multivariate model retained all those variables that both significantly improved fit of the model (*p*<0.05) and were independently associated with diarrhea (*p*<0.05).

We used a nested correlation structure for all general estimated equations analyses to account for at the first level the clustering of measures within the same village, and at the second level the clustering of repeated observations within households. We used SAS for Windows (PROC GENMOD) Version 9.1 (SAS Institute) for the general estimated equations modeling.

## Results

The evaluation team completed structured observations, baseline cross-sectional interviews, and initiated monthly surveillance in 347 households that did not receive the SHEWA-B intervention. Community monitors collected data on 465 children who lived in these 347 households for at least 1 mo. In the first month of diarrheal surveillance, there were 379 children <5 y living in these households. Their mean age was 19.2 mo. During 24 mo of follow-up, 66 children were born into these households, 20 children moved into the surveillance households, 24 children moved or dropped out, one child aged out, and 12 children died. The mean age of participating children after 24 mo was 37.7 mo. Among the 10,234 potential monthly child assessments from the time a child was first identified in a surveillance household, community monitors completed data collection for 9,897 (97%).

A third of the fathers and more than a quarter of the mothers had no formal education (Table 1). Over 90% owned their own home, but their median land holdings were quite small. Over 80% collected drinking water from shallow tube wells and three-quarters had access to an improved latrine. There was little difference in household characteristics between the 347 households included in the general analysis and the 102 households where fieldworkers observed a handwashing opportunity after defecation, though households where fieldworkers observed defecation owned somewhat more agricultural land. Within a household on the day of observation, handwashing practices before preparing food and after defecation varied, but fieldworkers noted the most thorough behavior commonly among all observations (Table 2).

**Table 2:** Distribution of handwashing by most thorough behavior observed versus all observations.

Handwashing Occasion	All Observations (%)	Most Thorough Behavior (%)
Before preparing food	585	281
Did not wash hands	296 (51)	102 (36)
Washed one hand with water only	133 (23)	75 (27)
Washed both hands with water only	153 (26)	101 (36)
Washed at least one hand with soap	3 (0.5)	3 (1.1)
After defecation	117	102
Did not wash hands	4 (3)	1 (1)
Washed one hand with water only	44 (38)	36 (35)
Washed both hands with water only	27 (23)	24 (24)
Washed at least one hand ash/mud	15 (13)	15 (15)
Washed at least one hand with soap	27 (23)	26 (25)

Caregivers reported that the child had diarrhea in the 48 h preceding the monthly interview in 947 (9.6%) of the 9,897 monthly assessments. In the bivariate analysis, household characteristics that were significantly associated with less child diarrhea included the mother or father having 7 or more years of education, a wealth index in the fourth quintile and ownership of a television, radio or mobile phone (Table 3). Children under the age of 2 y and observations during the first year of the surveillance were significantly more likely to have diarrhea (Table 3).

**Table 3:** Bivariate relationship between baseline characteristics and observed handwashing behaviors with subsequent diarrhea among children under age 5 y in the ensuing 24 mo.

Household Characteristics^a^	Person Months of Observation	*n* (%) Monthly Visits with This Exposure	*n* (%) Monthly Visits with Diarrhea	Adjusted Odds Ratio^b^	95% Confidence Interval^b^	*p*-Value^b^
Mother's education ≥7 y	9,897	3,327 (34)	241 (7.2)	0.81	0.67–0.98	0.031
Father's education ≥7 y	9,873^c^	3,341 (34)	234 (7.0)	0.72	0.54–0.95	0.018
Wealth quintile	9,897					
1 – baseline (poorest)		1,778 (18)	224 (12.6)	—	—	—
2		1,527 (15)	158 (10.4)	0.84	0.58–1.21	0.345
3		2,010 (20)	179 (8.9)	0.82	0.56–1.21	0.321
4		2,333 (24)	178 (7.6)	0.62	0.42–0.93	0.020
5		2,249 (23)	208 (9.2)	0.78	0.51–1.20	0.256
Owned radio	9,897	2,251 (23)	187 (8.3)	0.86	0.65–1.13	0.270
Owned television	9,897	2,934 (30)	225 (7.7)	0.84	0.72–0.98	0.031
Owned radio or television	9,897	4,261 (43)	333 (7.8)	0.82	0.68–1.00	0.048
Owned mobile phone	9,897	2,485 (25)	196 (7.9)	0.71	0.56–0.89	0.003
Owned water source	9,897	2,983 (29)	292 (10.1)	1.03	0.84–1.27	0.764
Owned toilet	9,897	5,092 (51)	514 (10.1)	1.04	0.83–1.30	0.736
Male child	9,897	4,776 (48)	466 (9.8)	1.07	0.90–1.28	0.429
Age <2 y	9,897	3,612 (36)	429 (11.9)	1.47	1.18–1.84	<0.001
Year 1 surveillance (versus Year 2)	9,897	4,747 (48)	563 (11.9)	1.72	1.27–2.34	<0.001
Exclusive breastfeeding last 24 h (among children age <2 1)	3,099	323 (10)	38 (11.8)	0.94	0.65–1.38	0.771
>1 child <5 y of age at home	9,897	3,615 (37)	377 (10.4)	1.18	0.93–1.51	0.177
**Structured Observation**						
Before preparing food						
Did not wash hands	8,023	2,957 (37)	370 (12.5)	—	—	—
Washed one hand with water only	8,023	2,187 (27)	182 (8.3)	0.79	0.59–1.07	0.133
Washed both hands with water only	8,023	2,797 (35)	192 (6.9)	0.70	0.52–0.94	0.0170
Washed at least one hand with soap	8,023	82 (1)	3 (3.7)	0.32	0.23–0.44	<0.001
Following handwashing optimal hand drying observed^d^	5,066	589 (12)	32 (5.4)	0.92	0.58–1.47	0.735
Before feeding a child						
Did not wash hands	8,093	4,070 (50)	416 (10.3)	—	—	—
Washed one hand with water only	8,093	3,102 (38)	302 (9.7)	0.86	0.69–1.08	0.192
Washed both hands with water only	8,093	685 (8)	62 (9.1)	1.19	0.85–1.68	0.314
Washed at least one hand with soap	8,093	236 (3)	19 (8.1)	0.63	0.20–1.31	0.221
Following handwashing optimal hand drying observed^d^	4,023	301 (7.5)	20 (6.6)	0.75	0.46–1.22	0.246
Before eating						
Did not wash hands	9,801	516 (5)	36 (7.0)	—	—	—
Washed one hand with water only	9,801	6,956 (75)	711 (10.2)	1.12	0.60–2.10	0.719
Washed both hands with water only	9,801	2,016 (22)	172 (8.5)	0.99	0.52–1.87	0.967
Washed at least one hand with soap	9,801	313 (3)	20 (6.4)	1.23	0.61–2.49	0.569
Following handwashing optimal hand drying observed^d^	9,285	312 (3)	18 (6)	0.77	0.48–1.23	0.273
After cleaning child's anus who had defecated						
Did not wash hands	3,913	273 (7)	37 (13.6)	—	—	—
Washed one hand with water only	3,913	1,186 (30)	110 (9.3)	1.32	0.59–2.92	0.497
Washed both hands with water only	3,913	1,165 (30)	135 (11.6)	1.65	0.73–3.77	0.231
Washed at least one hand ash/mud	3,913	305 (8)	20 (6.6)	1.14	0.45–2.89	0.779
Washed at least one hand with soap	3,913	984 (25)	102 (10.4)	1.58	0.56–4.42	0.383
Following handwashing optimal hand drying observed^d^	3,640	619 (17)	60 (9.7)	0.85	0.49–1.46	0.546
After defecation						
Did not wash hands	2,976	24 (1)	0 (0)	—	—	—
Washed one hand with water only^e^	2,976	1,029 (35)	135 (13)	—	—	—
Washed both hands with water only	2,976	711 (24)	76 (10.7)	0.77	0.46–1.29	0.321
Washed at least one hand ash/mud	2,976	431 (14)	30 (7.0)	0.62	0.34–1.14	0.124
Washed at least one hand with soap	2,976	781 (26)	47 (6.0)	0.45	0.26–0.77	0.003
Following handwashing optimal hand drying observed^d^	2,952	253 (9)	18 (7.1)	0.68	0.44–1.08	0.100

a When multiple handwash opportunities were observed in the same household, the household's handwashing behavior was classified on the basis of the most thorough handwashing behavior observed.

b Adjusted for repeated measures of the same child and village clustering.

c There were 24 fewer observations in the analysis with fathers' education, because there data were missing for one of the households.

d Optimal hand drying (air drying or drying with a clean towel) was compared with hands not dried or dried on dirty towel or clothing; this analysis was restricted to episodes where handwashing was observed.

e Washed one hand with water only was selected as the baseline category because too few people did not wash their hands at all to permit robust statistical evaluation.

Mothers reported at least some breast-feeding of their <1-y-old children in the preceding 24 h in 93% of monthly visits and reported exclusive breastfeeding of their <6-mo-old children in the preceding 24 h in 55% of monthly visits. Young children were both more likely to be breastfed and more likely to have diarrhea. After adjustment for age, neither any reported breastfeeding nor exclusive breast-feeding was associated with significantly less diarrhea.

Fieldworkers observed at least one opportunity to wash hands before preparing food in 281 (81%) of the households during structured observation. Handwashing before preparing food was associated with less diarrhea in the subsequent 2 y of follow-up (Table 3). In households where food was prepared without washing hands, children had diarrhea in 12.5% of monthly assessments compared with 8.3% in households where one hand was washed with water only, 6.9% where both hands were washed with water only, and 3.7% where at least one hand was washed with soap (Table 3). Food preparers commonly washed one or both hands with water only, but fieldworkers observed food preparers washing at least one hand with soap in only three households (1%).

Fieldworkers observed at least one opportunity to wash hands after defecation in 102 (29%) of the households during structured observation. Fieldworkers observed only a single household where residents never washed their hands after defecation. Handwashing with soap was much more common after defecation than before food preparation. In 25% of households, at least one household resident washed at least one hand with soap after defecation. Among the 27 observed episodes of handwashing with soap after defecation, in eight (30%) both hands were washed with soap. Children who lived in households where fieldworkers observed at least one hand washed with soap after defecation experienced substantially less diarrhea in the subsequent 2 y of follow-up compared with children who lived in households where only one hand was washed with water after defecation (Table 3).

The fieldworkers' observations of handwashing before feeding a child, before eating, and after cleaning a child's anus who had defecated were not associated with subsequent diarrhea (Table 3).

Among household residents observed washing hands, fieldworkers observed optimal hand drying, either allowing hands to air dry or drying hands on a clean towel, uncommonly, ranging from 3% before eating to 17% after cleaning a child's anus who had defecated. Children who lived in households where optimal hand drying was observed had somewhat less diarrhea than other children, but none of the observed differences were statistically significant.

In the multivariate analysis of structured observations before preparing food, washing both hands with water only and washing at least one hand with soap were both independently associated with significantly less diarrhea morbidity during 7,999 subsequent monthly assessments for diarrhea (Table 4). The number of months since initiating surveillance, child age less than 24 mo, father's education, and household ownership of a mobile phone were also independently associated with diarrhea, but the odds ratios for the structured observation of handwashing before preparing food in the multivariate analysis were nearly identical to the bivariate odds ratios.

**Table 4:** Multivariate analysis of observed handwashing behavior and subsequent diarrhea.

Characteristic	Adjusted Odds Ratio (95% Confidence Limit)^a^	*p*-Value
**Structured observation before preparing food** (*n* = 7,999)		
Before preparing food		
Did not wash hands – baseline	—	—
Washed one hand with water only	0.78 (0.57–1.05)	0.105
Washed both hands with water only	0.67 (0.51–0.889)	0.004
Washed at least one hand with soap	0.30 (0.19–0.47)	<0.001
Number of months since initiating surveillance	0.96 (0.94–0.98)	<0.001
Child age less than 24 mo	1.25 (1.01–1.55)	0.040
Father having education above primary level	0.72 (0.52–1.00)	0.052
Household owns a mobile phone	0.74 (0.57–0.97)	0.028
**Structured observation after defecation** (*n* = 2,952)		
After defecation		
Washed one hand with water only—baseline	—	—
Washed both hands with water only	0.79 (0.46–1.35)	0.389
Washed at least one hand ash/mud	0.63 (0.34–1.16)	0.135
Washed at least one hand with soap	0.45 (0.26–0.77)	0.004
Month since initiating surveillance	0.96 (0.94–0.98)	<0.001

a Odds ratio was calculated using a general estimated equations model that accounted for neighborhood clustering and repeated household sampling using a nested correlation structure.

In the multivariate analysis of structured observations after defecation, washing at least one hand with soap was independently associated with significantly less diarrhea in the 2,952 subsequent monthly assessments (Table 4). With a smaller sample size, the month since initiating surveillance was the only other factor independently associated with diarrhea. The odds ratios for the structured observation of handwashing after defecation in the multivariate analysis were nearly identical to the bivariate odds ratios.

## Discussion

In 50 villages across rural Bangladesh where fecal environmental contamination, undernutrition, and diarrhea are common, in those households where fieldworkers observed food preparers washing their hands before handling food, children under the age of 5 y experienced less diarrhea over the next 2 y compared with children living in households where food preparers did not wash their hands before preparing food. This observation suggests that before preparing food may be a particularly important time to promote handwashing [16].

Tomatoes, cucumbers, carrots, and various seasonal vegetables and greens are common components of meals in rural Bangladesh. Some of these vegetables are served raw but most are boiled and made into a curry that is commonly served with rice, the primary staple of the Bangladeshi diet. Many foods that are not further cooked, for example boiled root vegetables, fruits including bananas, and dried fish are often mashed and mixed by hand with spices and other ingredients during food preparation. Raw vegetables are commonly contaminated with pathogens and are a common vehicle for gastrointestinal pathogen transmission. Numerous outbreaks of gastroenteritis from a variety of pathogens have been traced to raw vegetables [17],[18]. The surface of raw cut lettuce and tomatoes is a hospitable environment for the growth of *Shigella* and *Salmonella* [19]–[21]. Similarly, there is considerable microbiological and epidemiological evidence that implicates cross-contamination of food as an important pathway for gastrointestinal pathogen transmission [22],[23]. Food that is inoculated with bacterial pathogens from contaminated hands may provide a nutrient rich environment that permits exponential growth for numerous pathogens [24]–[27]. The risk of diarrhea for many bacterial pathogens is proportional to the dose of the pathogens ingested [28],[29]. In several outbreaks of bacterial gastroenteritis, food that was contaminated several hours before serving was associated with high attack rates of gastroenteritis among persons who consumed it [30].

If persons preparing food did not wash their pathogen-contaminated hands before touching raw vegetables and rice, these foods may have become contaminated with gastrointestinal pathogens, which could subsequently multiply in a conducive growth environment before consumption. However, if the vegetables were cooked at a high enough temperature for a long enough time the pathogens would not survive and would not be transmitted. In future research, it would be useful to have fieldworkers specifically code the context of the handwashing opportunity around food preparation, so that the association between handwashing before handling raw vegetables and other foods that were subsequently cooked, handwashing before handling foods that were eaten raw, and handwashing before cross contaminating food that was not further cooked could be separately assessed.

In contrast to standard recommendations for handwashing that stress the central importance of using soap and specify detailed techniques for washing underneath fingernails, continuing lathering for over 20 s, and using either a clean towel or air drying to ensure effective handwashing [10], in this observational study, children who lived in households where food preparers practiced suboptimal handwashing (including briefly washing their hands with water alone) experienced significantly less diarrhea than children living in households where the food preparer did not wash hands at all. Fieldworkers did not directly measure the duration of handwashing with soap in this study, but in another study that used structured observation in urban Bangladesh to assess handwashing behavior and timed the duration of handwashing with soap with a stopwatch, the baseline mean duration of handwashing with soap was 5 s before preparing food and 11 s after defecation [31].

Although the benefits of handwashing with water alone observed in this evaluation conflict with standard recommendations, they are consistent with an older randomized controlled intervention study from urban Bangladesh. Stanton and Clemens used structured observation to observe handwashing behavior and noted an association between washing hands with or without soap and reported childhood diarrhea in a case control study in low-income urban communities in Dhaka Bangladesh [32]. In a subsequent intervention study in households that received the intervention, food preparers were significantly more likely to wash their hands with or without soap compared with food preparers in nonintervention households (49% versus 33%) [32]. Intervention households reported 26% less diarrhea than nonintervention households.

Microbiological studies demonstrate that washing hands with water alone reduces the concentration of various bacteria on hands [4],[33]–[35]. The reduction in these bacteria is generally less than the reduction in hand contamination following handwashing with soap [4],[33]–[36]. Field workers did not record the source of water used to wash hands, but the most common source of household water in rural Bangladesh is shallow tube wells. In other studies approximately 40% of water samples directly collected from shallow tube wells in Bangladesh were contaminated with fecal bacteria, though generally at a low-level of contamination [37],[38]. The present evaluation suggests that even the modest reduction in hand contamination achieved by washing with water alone reduces the risk of pathogen transmission at least during food preparation, albeit to a lesser degree than handwashing with soap.

The low proportion of households that followed recommended hand drying procedures suggests that substantial efforts would be required to change community habits to conform with hand drying recommendations. Since children living in households that practiced recommended hand drying behavior did not have significantly less diarrhea than other households, these data suggest that efforts to promote improved hand hygiene would be better focused on behaviors more strongly associated with child health, for example on handwashing before preparing food and after defecation, than on prescribing specific hand drying behavior.

People wash their hands more frequently when they know they are being observed [39]–[42]. In a previous study in rural Bangladesh that placed accelerometers within bars of soap to detect soap motion, the presence of an observer increased the frequency of soap motions consistent with handwashing by 35% [43]. Since Bangladeshi culture views adult feces as impure [8], social desirability bias may have increased observed handwashing with soap, especially after defecation. In the Bangladesh motion sensor study, residents of households with more education and who owned a mobile phone or watch were more likely to increase handwashing in the presence of an observer, and in the present study households with more education and those that owned mobile phones or televisions had less diarrhea [43]. Thus, an alternative interpretation of these observations is that the association between washing hands and subsequent childhood diarrhea is not causal. Rather, the observed reactive handwashing behavior might be an indicator of broader hygiene awareness that identified a subset of households that practiced a number of behaviors that contributed to less childhood diarrhea.

But there are two difficulties with this alternative interpretation. First, the strong association of handwashing with water alone before preparing food with diarrhea is less likely to result from social desirability bias, because there is no strong cultural norm for handwashing before preparing food. Indeed, only 1% of households washed hands with soap before preparing food. If household residents washed hands before preparing food because of social desirability bias that was then linked to other behaviors associated with less diarrhea, then we would also expect to find a significant association of diarrhea with handwashing before eating, before feeding a child, and after cleaning a child who defecated, associations that were not significant in this analysis. A second difficulty with attributing the observed associations to a theoretical unknown, unnamed, and unmeasured confounder is that the analysis implies that such a causal pathway for reduced diarrhea was independent of education, wealth, exclusive breastfeeding, and other evaluated household characteristics. An unmeasured personal or household characteristic that is so powerful that it dominates the relationship between handwashing and diarrhea, but is so elusive that we cannot even name it, seems a less likely explanation than pathogen contaminated hands and food, a biologically plausible explanation that invokes a pathway of gastrointestinal pathogen transmission repeatedly demonstrated in other contexts.

The observation in this evaluation that children living in households where residents washed their hands with soap after defecation had less diarrhea compared with children living in households where handwashing after defecation was less thorough is consistent with findings of previous intervention studies [1] and with handwashing interrupting the transmission of pathogens from the gastrointestinal tracts of household members to a susceptible child. The lack of a significant association of diarrhea with handwashing after cleaning a child's anus who defecated or handwashing before feeding a child or before eating also have plausible microbiological explanations. A child's gastrointestinal tract and immune system has already been exposed to the organisms in his/her own feces. Further exposure to these organisms is unlikely to cause clinical illness in the child. Unwashed hands can transmit pathogens to food, but when contaminated hands contact food at the time of eating or feeding, the dose of ingested pathogen is limited to the number of organisms that are passively transferred from hand to food. In contrast, when pathogens are transmitted to food items that are stored and not further cooked, bacterial pathogen populations may reproduce exponentially, resulting in a much higher dose of pathogen and a greater risk of diarrhea.

An important limitation of this study is that measuring handwashing on a single day risks misclassifying exposure. Among mothers in Burkina Faso, observed handwashing behavior after cleaning a child who had defecated was concordant with observations on a different day between 57% and 73% of the time [44]. This imperfect repeatability of handwashing assessments risks misclassifying exposure, which reduces the statistical power to identify associations. Such misclassification could explain why handwashing at some key times was not associated with less child diarrhea in this evaluation. However, handwashing in this evaluation was not classified on the basis of a single observation, but on the basis of the best behavior observed among multiple observations within the household (Table 2). Handwashing is a habitual behavior [2]. For example, in the Burkina Faso study, not washing hands on one occasion was significantly associated with subsequent behavior [44]. Importantly, even with reduced power from misclassification, the Bangladesh evaluation presented in this article identified associations between handwashing at two biologically plausible occasions with reduced prevalence of subsequent diarrhea.

A second limitation is that fieldworkers observed an opportunity to wash hands after defecation in only 29% of households. The resulting limited statistical power precluded a thorough assessment of the utility of washing hands after defecation with water only or with ash/mud, the contribution of other determinants of diarrhea, or a combined model that included both handwashing before preparing food and handwashing after defecation. However, even with limited power there was a strong association between handwashing with soap and less subsequent diarrhea, and the point estimates of the odds ratios are suggestive of less diarrhea for handwashing with water alone.

A third limitation is that different gastrointestinal pathogens have different routes of transmission within different contexts, which might limit the generalizability of this study. It is possible that transmission of gastrointestinal pathogens from hands to food during preparation is a less important route of transmission in other settings. Additionally, in settings where water to wash hands is more heavily contaminated with feces than available water in rural Bangladesh, washing hands with water alone may be less protective. However this evaluation was conducted in 50 rural villages in 26 districts across Bangladesh and Bangladesh is the eighth most populous country in the world, so the analysis is not identifying a highly isolated phenomenon. In an assessment of hygiene indicators in rural Nicaragua, washing hands before preparing food was the single hygiene indicator most strongly associated with child diarrhea [16]. Nevertheless, it would be useful to conduct similar evaluations in other contexts.

A fourth limitation is that the program evaluation was not designed to evaluate the hypothesis that observed handwashing behavior was associated with a change in the prevalence of subsequent diarrhea. Because this is a secondary analysis of the data, there is some risk of data mining to identify an interesting but ultimately not robust finding. However, we planned these data analyses at the time we designed the program evaluation. There was a dose effect between thoroughness of handwashing before preparing food and subsequent observed diarrhea and the associated *p*-values were <0.005.

An important flaw in this evaluation was that we did not have the protocol reviewed by an independent human study participant committee. The amount of time we asked from participants, the intensity of the interaction with the field team, and the use of these data to draw generalizable insights to improve global scientific understanding mean that the activity had substantial research components and should have been reviewed by an independent human study participant committee. The study team did implement standard procedures to minimize risks and harms to evaluation participants, but similar future evaluations should be reviewed by human study participant committees. Rigorous evaluations of large public health programs provide insights that can translate into improved programs that save lives and improve community health. However large public health programs in low-income countries often have extremely tight implementation schedules. Human study participant committees in low-resource settings may need to develop additional capacities to provide appropriate independent review more promptly for these type of evaluations.

Most people living in low-income settings have apparently concluded that following recommendations that require them to wash hands with soap ten, 20, or more times per day is not feasible [2]. The observations from this program evaluation suggest that to prevent childhood diarrhea the most important occasions for handwashing and the technique for effective handwashing differ from standard recommendations. Specifically, handwashing promotion programs in rural Bangladesh should not attempt to modify handwashing behavior at all five key times, but should focus primarily on handwashing after defecation and before food preparation. Because handwashing before food preparation is such a different context than after defecation, developing and evaluating strategies to promote handwashing before food preparation is an important area for future research.

The lower prevalence of childhood diarrhea seen in this evaluation among children living in households where residents washed hands with soap are consistent with the many intervention trials that demonstrate less childhood diarrhea in households where residents are encouraged to wash hands with soap [1]. The findings from this study, that children living in households where field workers observed food preparers washing their hands with water alone before preparing food had less diarrhea compared with children living in households where fieldworkers observed that food preparers did not wash their hands, suggest that promoting handwashing exclusively with soap may be unwarranted. Handwashing with water alone might be seen as a step on the handwashing ladder: handwashing with water is good; handwashing with soap is better. Additional controlled trials evaluating the effect on child health of interventions that include encouraging handwashing either with water alone or with soap and water would be particularly helpful to guide public health programs. More generally, research to develop and evaluate handwashing messages that account for the limited time and soap supplies available for low-income families, and are focused on those behaviors where there is the strongest evidence for a health benefit could help identify more effective strategies.

## Abbreviations

SHEWA-B: Sanitation, Hygiene Education and Water supply-Bangladesh
